# Retrospective analysis of 80 patients diagnosed with reversible cerebral vasoconstriction syndrome in the Helsinki Metropolitan Area

**DOI:** 10.1111/ene.16564

**Published:** 2025-01-29

**Authors:** Maria Girfanova, Nicolas Martinez‐Majander, Laura Mannismäki, Gerli Sibolt, Marjaana Tiainen, Sami Curtze

**Affiliations:** ^1^ Neurology University of Helsinki and Helsinki University Hospital Helsinki Finland

**Keywords:** RCVS, reversible cerebral vasoconstriction syndrome, stroke, subarachnoid haemorrhage, vasoconstriction haemorrhagic stroke

## Abstract

**Background and purpose:**

Up to 80% of patients diagnosed with reversible cerebral vasoconstriction syndrome (RCVS) experience complications such as ischaemic stroke, intracerebral or subarachnoid haemorrhage or posterior reversible encephalopathy syndrome. The aim was to evaluate the incidence of complications in patients diagnosed with RCVS in our clinic.

**Patients and methods:**

All adult patients (age >16 years) diagnosed with RCVS at the Helsinki University Central Hospital during the period between 1 January 2016 and 31 December 2022 were retrospectively identified. Medical and follow‐up data were collected from medical records.

**Results:**

Eighty patients diagnosed with RCVS were identified, of whom four patients had parenchymal lesions such as ischaemic stroke, intracerebral haemorrhage, posterior cerebral encephalopathy syndrome, subarachnoid haemorrhage or combinations thereof.

**Conclusion:**

The complication rate of RCVS is lower than in previously published cohorts. This may be related to better and earlier diagnostics and earlier withdrawal of possible triggers.

## INTRODUCTION

Reversible cerebral vasoconstriction syndrome (RCVS) has been increasingly recognized after proposal of the name in 2007 [1]. RCVS has been previously described since 1970 as isolated benign cerebral vasculitis, acute benign cerebral angiopathy, Call–Fleming syndrome, reversible cerebral segmental vasoconstriction or central nervous system (CNS) pseudovasculitis [2, 3, 4, 5, 6, 7, 8, 9, 10, 11].

RCVS typically presents with sudden and severe headaches, often described as thunderclap headache (TCH), which may be accompanied by focal neurological symptoms. Imaging techniques, such as computed tomography angiography (CTA) and magnetic resonance angiography (MRA), commonly reveal diffuse vasoconstriction of intracerebral arteries, which usually resolves within 3 months [3]. There is evidence that the vasoconstriction starts in the distal small arteries and progresses gradually to more proximal and larger arteries during the disease course. Centripetal propagation of vasoconstriction on magnetic resonance imaging (MRI) acquired at the time of TCH remission is an indicator of RCVS severity [4, 5, 6].

RCVS can be provoked by exposure to certain drugs, including sympathomimetic drugs [1], serotonergic drugs [7], immunosuppressants, immunomodulators, oral contraceptives, illicit drugs such as cocaine [8] and ecstasy, and other agents. Other conditions that are associated with RCVS are postpartum state [2, 9, 10], carotid stenting, open neurosurgical procedures, Takotsubo cardiomyopathy, thyrotoxicosis [1] and pheochromocytoma [9].

No randomized control trials have been conducted for treatment of RCVS. Current recommendations are based on expert opinion and observational data [10]. Empirical treatment with calcium channel blockers is frequently used. It is recommended to discontinue serotonergic and noradrenergic antidepressants, hormonal agents, immunosuppressive medications and immunomodulators. Additionally, exposure to other triggers should be avoided [1, 10]. Glucocorticoids and triptans should be avoided [11], whilst non‐steroidal anti‐inflammatory drugs and opioids can be used to reduce headaches [1].

Although patients diagnosed with RCVS usually have a favourable prognosis, RCVS can sometimes cause complications such as ischaemic stroke, parenchymal or subarachnoid haemorrhage (SAH) or oedema. Previous studies have revealed that up to 81% of patients may develop complications [1, 7, 12]. In a study on 2020 patients with RCVS, 17.1% had ischaemic stroke, 11% intracerebral haemorrhage (ICH), 32.7% SAH and 13.6% reversible brain oedema. Up to 29.7% of patients were discharged to a rehabilitation facility or nursing home after RCVS diagnosis [13]. A prospective study in a Korean population revealed that patients with idiopathic RCVS had a more benign disease course with a lower complication rate (12.5%) than patients diagnosed with secondary RCVS (complication rate 40.5%) [14]. Predictors of poor outcome include baseline clinical factors, such as advanced age and comorbid medical conditions, severe imaging findings (ICH, stroke, posterior reversible encephalopathy syndrome [PRES]) and exposure to glucocorticoid therapy [11, 13].

Based on our clinical observations, it appears that the frequency of parenchymal brain lesions in patients diagnosed with RCVS at our institution is <70%.

The aim of this study was to conduct a consecutive retrospective analysis to assess the complication rate of RCVS in a single comprehensive stroke centre setting.

## PATIENTS AND METHODS

All adult patients (>16 years old) diagnosed with RCVS (International Classification of Diseases (ICD) code I67.8, Other specified cerebrovascular diseases) at Helsinki University Hospital between 1 January 2016 and 31 December 2022 were retrospectively identified. Data were linked by unique personal identification numbers assigned to each case. Medical and follow‐up data were retrospectively collected from medical records. The authors verified all RCVS diagnoses by reviewing brain and vessel imaging, laboratory test results and medical records. Patients were excluded if the RCVS diagnosis was later revoked.

Patients diagnosed with definite or probable RCVS were included in the study.

RCVS diagnosis was based on key clinical features of secondary TCH or severe recurrent headache, cerebral vasoconstriction on imaging in at least two different arteries, and resolution of vasoconstriction within 3 months in the absence of primary angiitis of the CNS or aneurysmal SAH. Additionally, patients exhibiting the typical clinical presentation but with vasoconstriction localized to a single artery were included in the study. Furthermore, individuals presenting with focal neurological deficits, even in the absence of the characteristic headache, were considered if imaging (CTA or MRA) revealed vasoconstriction that subsequently resolved within the 3‐month timeframe. Although some of the recruited patients did not undergo control imaging for various reasons, they were not admitted later to hospital. Patients with missing follow‐up imaging were classified as probable RCVS, as the criterion of reversibility could not be verified.

The primary outcome measure was rate of complications during the disease course. The secondary outcome measure was clinical outcome at 3 months as assessed by the modified Rankin Scale (mRS) [15]. mRS assessment was performed retrospectively based on patient medical records.

Ischaemic stroke, ICH, SAH and transient ischaemic attack (TIA) diagnoses were based on American Heart Association/American Stroke Association (AHA/ASA) definitions. According to the AHA/ASA, CNS infarction is brain, spinal cord or retinal cell death attributable to ischaemia based on (1) pathological, imaging or other objective evidence of cerebral, spinal cord or retinal focal ischaemic injury in a defined vascular distribution or (2) clinical evidence of cerebral, spinal cord or retinal focal ischaemic injury based on symptoms persisting ≥24 h or until death and other aetiology excluded. TIA is defined as focal arterial ischaemia with transient symptoms lasting <24 h and without evidence of infarction by pathology or images. ICH is defined as a focal collection of blood within the brain parenchyma or ventricular system that is not due to trauma, including parenchymal haemorrhage after CNS infarction. Based on AHA/ASA definitions, SAH is a bleeding into the subarachnoid space [16]. No case of aneurysmatic SAH was found in this cohort based on vascular imaging and cerebrospinal fluid investigations.

Posterior reversible leukoencephalopathy syndrome was diagnosed by suitable clinical picture and characteristic MRI findings, including focal or confluent areas of increased signal on T2‐weighted and fluid‐attenuated inversion recovery images. Headache attributed to RCVS was diagnosed according to the diagnostic criteria of the latest International Classification of Headaches (ICHD‐3) [17]. Secondary TCH was defined as a severe head pain with abrupt onset, reaching maximum intensity in less than 1 min, lasting at least 5 min, and not better accounted for by another ICHD‐3 diagnosis [18]. The numerical rating scale from 0 (no pain) to 10 (worst pain ever experienced) is used routinely in our hospital for patient assessment.

‘Non‐smoker’ describes a person who has never smoked regularly; ‘ex‐smoker’ describes a person who previously smoked regularly but stopped >6 months before symptom onset. ‘Smoker’ describes a person who smoked regularly during symptom onset or discontinued smoking <6 months before symptom onset.

Our local protocol for RCVS workup includes computed tomography (CT)/CTA in the acute phase, which can be replaced with MRI/MRA in cases of neurological focal deficit, pregnancy, allergy or for other reasons. A lumbar puncture is recommended but not obligatory. All patients routinely undergo follow‐up imaging after 3 months, preferably with the same modality. The treatment protocol includes oral administration of calcium channel antagonists, such as amlodipine, lercanidipine or nifedipine.

The study was approved by the research board of the Neurocentre at Helsinki University Hospital. An ethical review for single‐centre retrospective analysis of data collected prospectively as a part of routine clinical care is not required at our institution.

Helsinki University Hospital is the only hospital providing neurological service to the Helsinki Metropolitan Area, with a catchment area of 1.6 million residents.

The authors declare that all supporting data are available within the article.

## RESULTS

Eighty patients diagnosed with RCVS in Helsinki University Hospital between 1 January 2016 and 31 December 2022 were identified; amongst these, 47 (59%) were female and 33 (41%) were male. Mean age was 41 years (range 17–74). Median time from first symptoms to admission was 6 days (range 0–31).

A total of 17 (21%) patients re‐contacted the emergency department (ED) at least once after RCVS diagnosis. Eight (10%) patients contacted the ED due to headaches that were not TCH, four (5%) patients sought treatment for postdural puncture headache and three (4%) patients for TCH. One patient was admitted to the ED due to sensory deficit and one due to nifedipine side effects. The reason for admitting one patient to the ED was unknown. Patient, pain and trigger characteristics are provided in Table 1. The most common trigger for TCH was sexual intercourse.

**TABLE 1:** Patients' characteristics.

	*N*	%
All patients	80	100
Female	47	59%
Male	33	41%
Triggers		
Sexual intercourse	28	35%
Workout	16	20%
Light physical activity	11	14%
Vasoactive medications, medication started within 3 months before symptom onset	4	5%
Alcohol consumption, hangover	4	5%
Driving	2	2.5%
Infections	2	2.5%
Emotional stress	2	2.5%
Diving	1	1.3%
Drugs and illicit substances	1	1.3%
Information not available	7	8.8%
Trigger not identified	6	7.5%
Symptoms		
Isolated TCH	71	89%
Non‐TCH transient headache	3	3.75%
TIA and non‐TCH headache	2	2.5%
TIA without headache	2	2.5%
Persistent headache	1	1.25%
TCH and TIA (aphasia)	1	1.25%
Smoking		
Non‐smoker	35	44%
Ex‐smoker	26	33%
Smoker	13	16%
History of previous headaches		
Migraine, undefined	10	12.5%
Migraine with aura	6	7.5%
Migraine without aura	6	7.5%
No history of migraine	58	72.5%
Chronic headaches NAS	7	8.8%
Cluster headaches	2	2.5%
Tension neck	2	2.5%
Follow up		
mRS (3 months) 0–2	69	86%
Alive, but insufficient data for mRS estimation	11	14%
Fatal outcome (*n* = 80)	0	0%

Abbreviations: mRS, modified Rankin Scale; NAS, Not Adequately Specified; TCH, thunderclap headache; TIA, transient ischaemic attack.

A total of 71 (89%) patients were admitted to the ED due to isolated TCH. For the other nine patients, one experienced transient aphasia and TCH, three experienced transient headache that was not described as a TCH, two patients experienced TIA associated with non‐TCH headache, two patients experienced TIA without headache, and one patient was admitted to the ED due to persistent headache that was not described as TCH. Amongst the patients who experienced TCH before admission, 15 (19%) patients had a single TCH episode, 15 (19%) experienced two episodes of TCH and 42 (53%) reported three or more episodes.

Thirty‐six (45%) patients were discharged from the ED and 41 (51%) were admitted to the neurological department. Three (4%) patients were discharged initially but then admitted to the neurological department within a few days. Two of these patients were admitted due to severe postdural puncture headache and one due to recurrent severe TCH.

### Imaging

Imaging results are presented in Tables 2, 3, 4. CT/CTA was performed as first‐choice investigation for 63 (79%) patients. MRI/MRA was performed as first‐choice investigation or as confirmation for RCVS diagnosis after CT/CTA during the acute phase for 48 (60%) patients. Thirty‐one (39%) patients were investigated with both CT/CTA and MRI/MRA during the acute phase.

**TABLE 2:** Imaging findings of patients diagnosed with RCVS, parenchymal lesions and/or SAH.

	CT finding	MRI finding	Age	Sex	Trigger	Delay from TCH onset to admission
Patient 1	Suspicion of ischaemic stroke	Normal	40	F	Workout	21 days
Patient 2	ICH and SAH	ICH and SAH	35	M	N/A	1 day
Patient 3	SAH	SAH, PRES	60	F	Light physical activity	1 day
Patient 4	SAH	SAH, ICH, PRES	74	F	N/A	No TCH history
Patient 5	SAH	Not performed	38	F	N/A	29 days

Abbreviations: CT, computed tomography; ICH, intracerebral haemorrhage; MRI, magnetic resonance imaging; PRES, posterior reversible encephalopathy syndrome; RCVS, reversible cerebral vasoconstriction syndrome; SAH, subarachnoid haemorrhage; TCH, thunderclap headache.

**TABLE 3:** CTA findings.

	*N*	%		
All patients	80	100%		
CTA as a first‐choice investigation modality	63	79%		
Vasoconstriction on the first investigation	53	84%		
1 vessel	8	15%		
2–4 vessels	19	36%		
>4 vessels	26	49%		
Vasoconstriction severity (CTA)				
Mild	17	32%		
Moderate	36	68%		
Circulation (CTA)				
Anterior	13	25%		
Posterior	9	17%		
Both	31	58%		

Abbreviation: CTA, computed tomography angiography.

**TABLE 4:** MRA findings.

	N	%		
All patients	80	100%		
MRA as a first‐choice investigation modality	48	60%		
Vasoconstriction on the first investigation	43	90%		
1 vessel	7	16%		
2–4 vessels	20	47%		
>4 vessels	15	35%		
Vasoconstriction severity (MRA)				
Mild	17	40%		
Moderate	26	60%		
Circulation (MRA)				
Anterior	9	21%		
Posterior	7	16%		
Both	26	60%		

Abbreviation: MRA, magnetic resonance angiography.

Four (5%) patients with parenchymal lesions or SAH were identified amongst our cohort. Based on a CT scan, combined ICH and SAH were identified in one (1%) patient and isolated SAH was identified in three (4%) patients. Although ischaemic stroke was suspected in one (1%) patient, this was not verified on subsequent MRI. Imaging findings of CT and MRI are presented in Table 1. Two patients had PRES based on MRI findings. The patients admitted due to neurological focal deficits also had lesions on CT/MRI.

Vasoconstriction was visible in 53 patients investigated with CTA with vasospasms only in a single vessel in eight (10%) patients, vasoconstriction in two to four vessels in 19 (24%) patients and vasoconstriction in multiple vessels (more than four) in 26 (33%) patients. Our neuroradiologist graded ‘mild’ vasoconstriction in 17 (32%) patients and ‘moderate’ in 36 (68%) patients. Only posterior circulation was affected in nine (17%) patients, anterior circulation was affected in 13 (25%) patients, and both anterior and posterior circulation were affected in 31 (58%) patients.

Vasoconstriction was visible in 42 patients investigated with MRA. Seven (17%) patients had vasoconstriction only in a single vessel and 20 (48%) patients had vasoconstriction in two to four vessels. Vasoconstriction was observed in multiple vessels in 15 (36%) patients. Mild vasoconstriction was observed in 16 (38%) patients and moderate in 26 (62%) patients. Only posterior circulation was affected in seven (17%) patients, anterior in nine (21%) patients, and both anterior and posterior circulation were affected in 26 (62%) patients.

Follow‐up imaging of vessel status was available in 70 (88%) patients and missing in 10 (12%) patients for various reasons. CTA was used as follow‐up imaging in 22 (28%) patients. Vasoconstriction worsened in two (9%) patients, resolved in 17 (77%) patients and improved in three (14%) patients in 3 months. Vasoconstriction resolved in all patients during additional follow‐up scans. The internal carotid artery was not affected in all patients.

MRA was used as follow‐up imaging in 48 patients (60%). Vasoconstriction resolved in 43 (90%) patients, improved in four (8%) patients and initially worsened in one (2%) patient. Vasoconstriction resolved in all patients according to additional follow‐up scans performed after 2 months. Two patients (3%) were imaged with both CTA and MRA.

### Laboratory tests

Lumbar puncture was performed in 61 (76%) patients. Pleocytosis with a white blood cell level of 64 E9/l was observed in one patient and was explained by SAH and ICH. Cerebrospinal fluid was normal in other patients.

### Treatment

A total of 79 (99%) patients received treatment with calcium channel blockers. Amlodipine was prescribed for four (5%) patients, felodipine for two (3%) patients, lercanidipine for three (4%) patients, nicardipine infusion following oral amlodipine for one (1%) patient, nifedipine for 53 (66%) patients, nilvadipine for one (1%) patient, nimodipine for 12 (15%) patients and verapamil for three (4%) patients. One (1%) patient received only symptomatic treatment with painkillers and antiemetics.

Median treatment duration was 12 weeks (range 1–36). Length of treatment was unknown for three (4%) patients. Four (5%) patients continued taking calcium channel blockers after resolution of vasoconstriction due to different reasons, such as arterial hypertension (three patients) and eclampsia (one patient).

### Risk factors and triggers

Amongst all the patients diagnosed with RCVS, 35 (44%) patients were non‐smokers, 26 (33%) current smokers and 13 (16%) were ex‐smokers. There was no information about tobacco use in six (8%) patients.

Three (4%) patients started using a nasal decongestant shortly before symptom onset. One patient used triptans for migraine headaches almost daily for several weeks before symptom onset. One patient had TCH about 2 weeks after consuming cocaine. There were no mentions of new medications or drugs started within 3 months before symptom onset in other patients. Although one patient was undergoing cancer therapy, cytostatic treatment with bevacizumab was ongoing for months before first RCVS symptoms.

Twenty‐two (28%) patients had a previous diagnosis of migraine, 10 were diagnosed with unspecified migraine, six with migraine with aura and six with migraine without aura. Fifty‐eight (73%) patients had no previous history of migraine. Seven (9%) patients were previously suffering from chronic headaches that did not fulfil migraine or other specific headache criteria (chronic headache NOS (not otherwise specified)). Two (3%) patients had previous experience of TCH that was not caused by aneurysm rupture or RCVS. Two (3%) patients were previously diagnosed with cluster headaches and two (3%) patients were diagnosed with tension headaches. Fourteen (18%) patients had history of depression, anxiety or both before symptom onset.

One patient had a previous diagnosis of RCVS before current symptom onset.

### Follow‐up

Sixty‐seven (84%) patients had an mRS of 0 at the 3‐month follow‐up. Two (3%) patients had an mRS of 2. For 11 (14%) patients, mRS evaluation was not possible due to incomplete medical records. However, according to the patient database, all patients were alive at the time of writing. Only one patient was readmitted to the ED a few years after suffering from RCVS due to a headache. This patient was not diagnosed with relapse of RCVS and the headache was most probably due to chronic migraine, Horton's neuralgia or both.

One patient was graded mRS 2 because of a lesion in the occipital lobe that caused inability to drive a car (unable to perform normal activities). Another patient who was graded mRS 2 initially had transient hemiparesis and aphasia; however, symptom progression subsequently occurred.

New diagnoses detected during the follow‐up were arterial hypertension for 10 (13%) patients, anxiety and depression for two (3%) patients, primary hyperaldosteronism for one patient, cluster headaches for one patient, and Factor V Leiden mutation for one patient. One patient was diagnosed with pheochromocytoma 15 months later and later had a hypertensive crisis accompanied by PRES and brain and spinal cord infarctions.

## DISCUSSION

According to previous studies, the incidence of parenchymal changes, SAH or both amongst patients diagnosed with RCVS varies considerably (12.5%–80%) [1, 3, 4, 12, 14, 19, 20]. In our clinical experience, most patients diagnosed with RCVS in our hospital do not need admission to the neurological ward and have a benign disease course. In this retrospective study 80 patients diagnosed with RCVS were identified.

According to the literature, the female preponderance varies from 1.8:1 to 10.2:1, which is higher than this study (1.4:1) but consistent with previous reports [4].

Most patients in our cohort had an identifiable trigger (84%). The most common trigger was sexual intercourse (35%), which is consistent with the most common triggers in previous reports [12].

Amongst our cohort of 80 patients, only four patients (5%) were diagnosed with brain parenchymal lesions, SAH or both, which is much lower than previously reported [1, 4, 20]. The most frequent complication of RCVS was SAH (5%), which is consistent with previous reports [4, 20]. PRES was identified in only two (2.5%) patients in our cohort, which is lower than previously reported [4]. None of our patients were diagnosed with ischaemic stroke; this is lower than in previous studies, where 7%–9% of patients diagnosed with RCVS had ischaemic stroke [4].

The low incidence of parenchymal lesions amongst patients diagnosed with RCVS in our cohort may be due to more frequent vessel imaging of patients admitted to the ED due to TCH and therefore resulting in diagnosis of milder cases of vasoconstriction. It is also possible that improved understanding of the disease and its risk factors led to avoidance of potential triggers, resulting in faster resolution of vasoconstriction than in previous cohorts.

Compared with previously published studies, fewer patients in our cohort experienced RCVS due to vasoactive drug exposure (41.5%–60% vs. 5%) [3, 4]. In our cohort, the median time from symptom onset to admission to the ED was 6 days. The short median time from symptom onset to withdrawal of triggers and initiation of treatment may explain the low rate of ischaemic complications, as ischemic lesions develop at around day 12 ± 3 from symptom onset [4].

MRI/MRA was performed on 60% of the patients in our cohort, which may partially account for the low complication rate observed. In a study by Singhal et al., most patients underwent both CT and MRI; however, the exact number was not specified. Their findings indicated that up to 81% of patients developed brain lesions [19]. Conversely, in a prospective study from Korea, although 129 out of 138 patients (93.5%) underwent MRI, the complication rate remained relatively low, with only 15.4% of patients affected [14].

Consistent with the literature, the clinical outcome amongst our patients was good with mRS 0–2 in 86% of the patients. An initially transient focal deficit reoccurred as a permanent deficit in two patients who therefore scored a 2 on the mRS. For 14% of the patients, the 3‐month mRS could not be evaluated due to missing documentation. However, according to the data, all patients were alive at the time of writing. It is also worth noting that, if these patients had experienced complications, they would most probably be admitted to our hospital.

The retrospective nature of this study has limitations, as it relies on past medical records that may not capture all relevant information accurately. Additionally, there is a possibility that some patients diagnosed with parenchymal changes due to RCVS may lack the corresponding RCVS ICD code in their records, potentially leading to underrepresentation of cases. The TIA diagnosis was made by an experienced neurologist and a patient chart review was performed by an experienced stroke neurologist for this study. However, there is a risk of misdiagnosis due to the nature of TIA. Patients with probable RCVS have been included in our study, which introduces some limitations. However, previous studies have also typically included probable RCVS cases, comprising up to 25% of the cohort [14].

Our study benefits from using the only hospital in the Helsinki Metropolitan Area offering specialized neurological treatment. With a dedicated neurological ED led by neurologists, high‐quality care and expertise are ensured in diagnosing conditions like RCVS. Moreover, our standard practice of conducting imaging studies, including angiography, for all patients admitted with TCH provides comprehensive data for analysis, which improves the reliability of our findings.

## CONCLUSION

A lower complication rate was observed amongst patients diagnosed with RCVS compared with previous reports. This may be due to the fact that initial workup of TCH in our clinic includes routine vascular imaging and thus milder cases are diagnosed earlier. However, it is crucial to search for underlying causes of RCVS, as potentially treatable conditions, such as pheochromocytoma, may be found. Furthermore, the efficacy of calcium channel blockers in managing RCVS warrants further investigation through randomized controlled trials in the future.

## AUTHOR CONTRIBUTIONS

**Maria Girfanova:** Writing – review and editing; writing – original draft; investigation. **Nicolas Martinez‐Majander:** Writing – review and editing. **Laura Mannismäki:** Writing – review and editing. **Gerli Sibolt:** Writing – review and editing. **Marjaana Tiainen:** Writing – review and editing. **Sami Curtze:** Conceptualization; methodology; data curation; supervision; project administration; writing – review and editing; funding acquisition; resources.

## CONFLICT OF INTEREST STATEMENT

The authors declare no conflict of interest.

## Data Availability

The data that supports the findings of this study are available in the supplementary material of this article.
